# Cementoma of the calcaneus: a case report

**DOI:** 10.1186/s12891-017-1460-1

**Published:** 2017-03-14

**Authors:** Xiaona Li, Zhigang Peng, Mahrukh Latif, Abhinav Kumar, Wei Chen, Zekun Zhang

**Affiliations:** 1 Department of Radiology, The Third Hospital of Hebei Medical University, Shijiazhuang, China; 2 Hebei Province Biomechanical Key Laborary of Orthopedics, Ziqiang road 139, Shijiazhuang, Hebei 050051 China

**Keywords:** Cementoma, Calcaneus, Radiography, Computerized tomography

## Abstract

**Background:**

The cementoma is a common disease of the dental root apex, which generally occurs in the maxilla and the mandible, but the cementoma occurring in the long bone is rare. Moreover, the incidence of cementoma in the calcaneus is extremely infrequent.

**Case presentation:**

The present study reports an unusual case of a 19-year-old girl, who complained of pain in the left heel. Subsequent radiographs and computed tomography (CT) were used in the diagnosis. The imaging features of the lesion included a radiopaque matrix and radiolucent tissue, particularly an arc-shaped fat band. An excisional biopsy was performed. Histopathological examination confirmed the diagnosis of cementoma in the calcaneus. After the operation, the patient was followed up without recurrence.

**Conclusions:**

Imaging examination plays an important role in the differential diagnosis of cementoma of the calcaneus.

## Background

Extragnathic cementoma of long bone as a benign bone neoplasm is very rare, which was reported to mainly locate in the lower limb in the literature [1–4]. In 1969, Friedman and Goldman first reported cementoma of long bones [5]. To 2005, there were 10 cases reports about this tumor of long bones in total [4].

We report an extragnathic cementoma of the calcaneus in a 19-year-old woman, who presented with intermittent pain at the left heel half a year ago. The imaging features including radiography and computerized tomography (CT) are described in our study. To the best of our knowledge, no report has previously introduced this tumor occurring in the calcaneus. Written informed consent was obtained from the patient.

## Case presentation

**Fig. 1:**
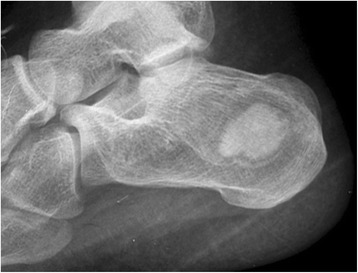
Lateral radiograph of the left calcaneum. An expansive, partially osteolytic lesion is displayed, with well-defined margins, and an eccentric calcified matrix

**Fig. 2:**
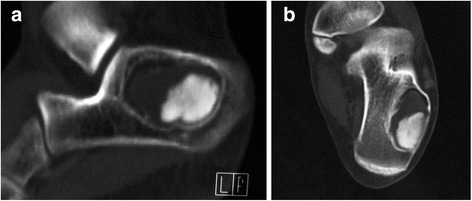
Computed tomography (CT) scans of the calcaneum. **a** Saggital reconstruction image and (**b**) coronal reconstruction image show an osteolytic lesion with sclerotic margin and an eccentric focus of matrix calcification adjacent to soft tissue dense with an arc-shaped fat band

A 19-year-old woman presented with intermittent pain over a period of 6 months in the left heel, which was severe after activity and relieved after taking rest. There was no history of trauma to the left foot. She visited our hospital for treatment and diagnosis because of worsening pain in last 10 days. The pain was limited to the heel. Tenderness was noted in the posterior part of the calcaneus. No subcutaneous mass was palpated. Normal range of motion was demonstrated in the ankle. Radiograph of the left foot revealed a well-defined osteolytic lesion with marginal sclerosis, involving the calcaneal tuberosity. The size of the lesion was 3.5 × 2.5 × 2.0 cm (Fig. 1). An irregular radiopaque mass was observed in this elliptic lesion. For further radiological examination of the lesion, CT scan of the left foot was chosen. An osteolytic lesion was clearly showed in the calcaneal tuberosity. Expansion with a thin peripheral cortex was detected, but not breaking through the calcaneus. There was no swelling in the surrounding soft tissue. A homogenous hyperdense deposit with a size of 2.5 × 2.0 × 1.0 cm was seen in the lesion. The sagittal and transversal images showed the radiopaque deposit was surrounded by the radiolucent tissue. An arc-shaped fat band was laid to the front of the radiolucent tissue. The radiolucent tissue presented the soft tissue dense which is the tumor tissue and CT value was 30.6 Hounsfield Units (HU). The irregular mass was 712.7 HU which was the calcified structure (Fig. 2b, c). Taken together, the lesion was initially considered as calcifying solitary bone cyst, intra-osseous lipoma, enchondroma and osteoblastoma. Extragnathic cementoma was hypothesized and surgical excision was recommended.

**Fig. 3:**
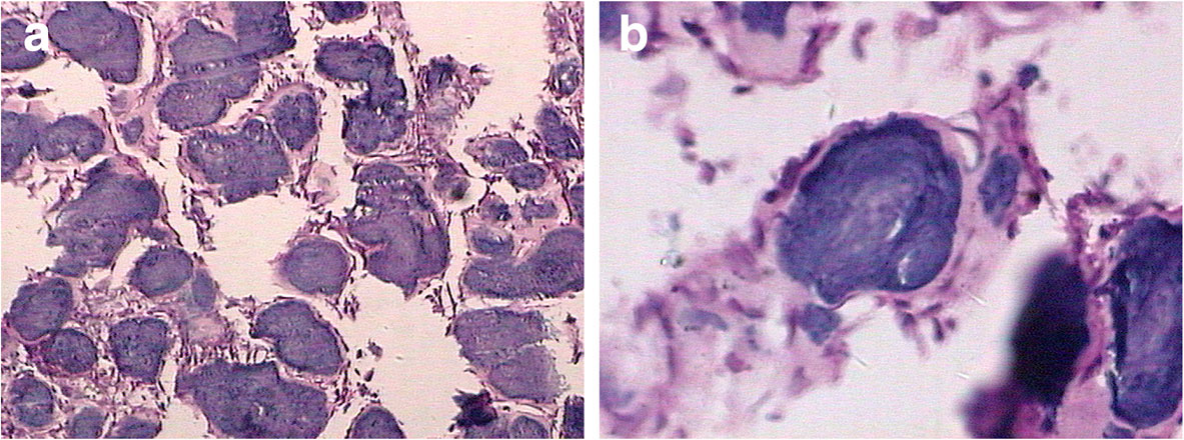
Pathologic specimen (A: HE × 100 and B: HE × 400) of the lesion. **a** A fibrous tissue with almost acellular stroma, containing irregularly shaped spherules is seen. **b** Large deposits of cementum-like material are seen in the spherules

During operation, the cortex of the affected bone was found to be thin. Granulation with fibrous tissue was observed in the lesion. Calcified sample particles mixed together were also seen. A large stiff sclerotic mass was took out of the calcaneus and appeared pinkish-grey or grey. An excisional biopsy was performed. Histopathological examination revealed there was abundant fibrous tissue containing calcified spherules and few cells in the excised specimens. Cementum-like material was found within the calcified spherules, which irregularly formed acellular trabeculae. Some cementum corpuscles were mixed together (Fig. 3a, b). Thus a diagnosis of cementoma of the calcaneus was confirmed.

**Fig. 4:**
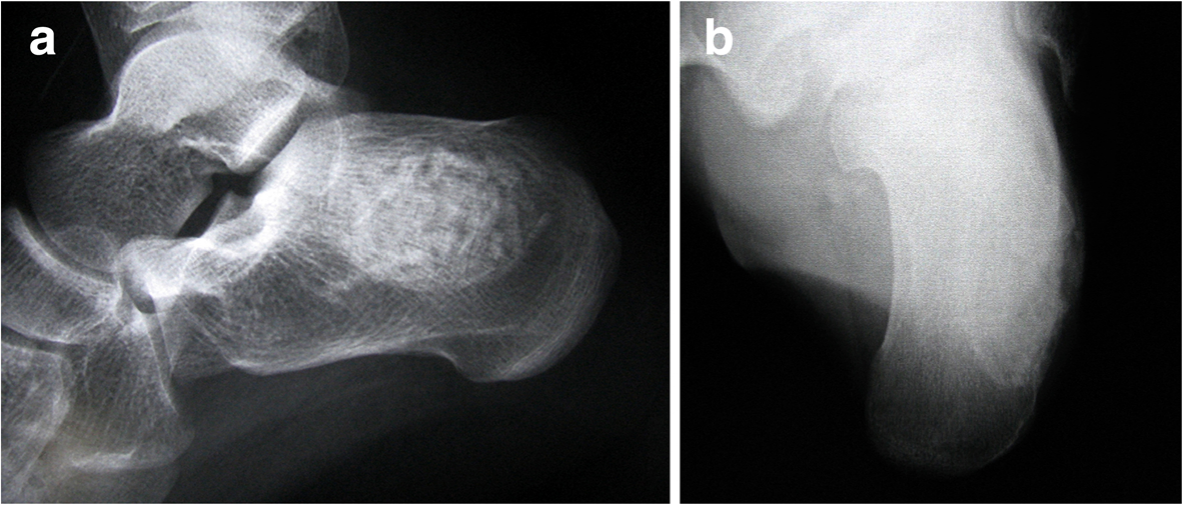
Post-operation radiograph of the calcaneum. **a** Lateral image and (**b**) axial image show the surgical cavity is filled with bone grafts

The patient was treated by curettage, bone grafting and bone packing with the iliac crest in the same side. There was no evidence of recurrence at the 2-year follow-up (Fig. 4a, b).

## Discussion

According to the World Health Organization (WHO), the classification of odontogenic tumor keeps changing [6, 7]. Cementoma, as a common benign odontogenic tumor, generally occurs in the maxilla and the mandible [4]. Cementoma occurring in the long bone is the rare bone lesion [1, 8]. There is a controversy in the cementoma located in the long bones, due to the reason that many authors do not accept the existence of true cementum outside the gnathic region. Mirra et al. [8] thought cementoma of long bone is merely a form of simple bone cysts. In 1985, Adler [2] found there was calcified cementum-like material accompanied by few fibroblasts in the solitary bone cysts. They thought cementoma was originally bone cysts which calcified steadily with age [2]. This tumor in the long bone has been reported in three separate types of lesions [3], including a simple bone cyst with associated cementum-like particles in the cyst lining, calcified cementum such as is present in our patient, cementifying fibroma. To date, there are only 10 reported cases of cementomas occurring in the extragnathic long bones [2, 4, 5]. In addition, Kolar et al. [1] first reported cementoma of the metacarpus occurring in the short tubular bone. So far the case of the calcaneus has not been reported in the literature. The present case which belongs to extragnathic irregular bones is the first report of cementoma occurring in the calcaneus.

In the English literature, the radiographic appearances in the cementomas of the long bones were quite similar [4]. The lesions were mostly located in the center of the medullary cavity, and mainly diaphyseal-metaphysis [1]. Most tumors appeared as well-defined, expansive, radiolucent lesions with radiopaque matrix [4]. The pathologic specimen consists of cementum corpuscle as is present in the cementomas of the jaw bones [5].

This new observation of this rare entity found in our patient differs from others by its localization (calcaneus medullary, not long bone). The radiographic and microscopic appearance of the cementoma found in the present patient was consistent with the findings reported by previous authors. On radiograph, the lesion presented a cavity surrounded by an incomplete sclerotic rim and a radiopaque mass was present within it. On CT, it was cortical expansive, and had a well defined sclerotic border, peripheral radiolucency, and an amorphous eccentric focus of matrix calcification. These radiographic features resembled those of their maxillary counterparts and other previously reported lesions of the long bones containing cementum or cementum-like material [1, 9, 10]. One noteworthy feature of the lesion from the imaging studies should be mentioned that is the peripheral radiolucency seen on CT images around the calcified matrix. Some reports suggested that the peripheral radiolucency tissue was in a transitional stage [11], or the absence of calcified spherules within the fibrous tissue on the periphery of the lesion [4]. However, it is a pity that we could not find any literature to support this manifestation of radiolucency of tumor tissue with an arc-shaped fat band. Therefore, we present this rare case as an effort to further characterize the lesion and to facilitate the diagnosis of similar cases.

Radiologically, cementoma of extragnathic bone has been classified into three stages [12–14]: osteolytic stage with cystic low density, cementum formation stage with mass shadow in the lesion and mature stage. Characteristic mature stage is also called inactive stage which has high clinical value, such as our case.

The radiographic differential diagnosis of cementoma of the calcaneus includes enchondroma [15], osteoblastoma [16], bone infarction [17], intra-osseous lipoma [18] and calcifying solitary bone cyst [19]. Enchondroma usually grows in the center of the medullary cavity of calcaneus with an irregular bone protuberance with a wide base and contains characteristic chondroid-like calcifications without adjacent sclerotic rim [4, 15]. However cementoma of the calcaneus displays a well-defined, sclerotic rim surrounding the lesion. Osteoblastoma shows a clear thin shell-shaped calcifications surrounding the lesion with low density bone expansion and destruction and soft tissue infiltration [20]. Bone infarction usually displays map-shaped lesion in the center of the calcaneus [17]. The lytic areas are permeated by focal or diffuse medullary osteosclerosis but not affected the cortex. Intra-osseous lipoma [18] shows the lesion divided by fibrous septa and an almost uniform density of fat. CT examination is a helpful method to rule it out. Generally, it is difficult to distinguish between diagnosis of calcifying solitary bone cyst and cementoma [19], especially for the cementum of osteolytic stage. Calcifying solitary bone cyst is a changed solitary bone cyst, and associated with symptoms such as swelling, deformation and pathological fracture [10]. Calcifying solitary bone cyst displays the lesion, a fluid filled structure, with cortical thinning or destruction of the calcaneus with small or multiple foci calcifications. In cementoma, however, the calcification is an amorphous central or eccentric cementum-like matrix.

### Limitations of the study

Since this study was a retrospective study, no MR examination was done and intraoperative photos were not acquired.

## Conclusions

We first present a rare case of cementoma of the calcaneus. When compared with dental cementoma, cementoma of the extragnathic bone is rare, mainly occurring in the long bone. The radiologic imaging features of this lesion, including the radiopague deposit and radiolucent tissue with an arc-shaped fat band should be concerned by the radiologists. When cementum corpuscle was detected, the diagnosis of cementoma should be considered. We report this case and hope to provide informative data that confirm that the cementoma of the extragnathic bone exists as a distinct entity.

## Abbreviations

CT: Computered tomography
HU: Hounsfield Units
WHO: World Health Organization
